# Acute kidney injury and the risk of mortality in patients with methanol intoxication

**DOI:** 10.1186/s12882-019-1404-0

**Published:** 2019-06-06

**Authors:** Shu-Ting Chang, Yu-Ting Wang, Yi-Chou Hou, I-Kuan Wang, Hsiang-Hsi Hong, Cheng-Hao Weng, Wen-Hung Huang, Ching-Wei Hsu, Tzung-Hai Yen

**Affiliations:** 1 Department of Physical Medicine and Rehabilitation, Chang Gung Memorial Hospital, Linkou, Taiwan; 2 Department of Pediatrics, Taipei Municipal Wan Fang Hospital, Taipei, Taiwan; 3 Division of Nephrology, Department of Internal Medicine, Cardinal Tien Hospital and School of Medicine, Fu-Jen Catholic University, New Taipei City, Taiwan; 4 Department of Nephrology, China Medical University Hospital and College of Medicine, China Medical University, Taichung, Taiwan; 5 Department of Periodontics, Chang Gung Memorial Hospital and Chang Gung University, Linkou, Taiwan; 6 Department of Nephrology and Clinical Poison Center, Chang Gung Memorial Hospital and College of Medicine, Chang Gung University, Linkou, Taiwan; 7 Kidney Research Center, Chang Gung Memorial Hospital, Linkou, Taiwan; 8 Center for Tissue Engineering, Chang Gung Memorial Hospital, Linkou, Taiwan; 9 Department of Nephrology, Chang Gung Memorial Hospital, 199 Tung Hwa North Road, Taipei, 105 Taiwan

**Keywords:** Methanol, Ethanol, Mortality, Acute kidney injury, Fomepizole, Haemodialysis

## Abstract

**Background:**

Methanol poisoning is a serious public health issue in developing countries, but few data are available in the literature on acute kidney injury (AKI) after methanol intoxication.

**Methods:**

This study examined the clinical features, spectrum and outcomes of AKI in patients with methanol intoxication and evaluated the predictors of mortality after methanol intoxication. A total of 50 patients with methanol intoxication were seen at Chang Gung Memorial Hospital between 2000 and 2013. Patients were grouped according to the status of renal damage as AKI (*n* = 33) or non-AKI (*n* = 19). Demographic, clinical, laboratory, and mortality data were obtained for analysis.

**Results:**

Most patients were middle-aged (47.8 ± 14.9 years), predominantly male (74.0%), and habitual alcohol consumers (70.0%). Most incidents were oral exposures (96.0%) and unintentional (66.0%). Two (4.0%) patients attempted suicide by intravenous injection of methanol. Five (10.0%) patients suffered methanol intoxication after ingestion of methomyl pesticide that contained methanol as a solvent. Compared to non-AKI patients, the AKI patients were older (50.9 ± 13.7 versus 41.6 ± 15.6 years, *P* = 0.034), predominantly male (90.9% versus 42.8%, *P* = 0.000), more habitual alcohol users (84.8% versus 41.2%, *P* = 0.001) and had more unintentional exposures (82.8% versus 35.3%, *P* = 0.001). Furthermore, there was a higher incidence of respiratory failure (63.6% versus 29.4%, *P* = 0.022) in the AKI group than in the non-AKI group, respectively. The laboratory studies revealed that the AKI patients suffered from more severe metabolic acidosis than the non-AKI patients. By the end of this study, 13 (39.5%) AKI patients and 1 (5.9%) non-AKI patient had died. The overall in-hospital hospital mortality rate was 28%. In a multivariate binary logistic regression model, it was demonstrated that AKI (odds ratio 19.670, confidence interval 1.026–377.008, *P* = 0.048) and Glasgow coma scale score (odds ratio 1.370, confidence interval 1.079–1.739, *P* = 0.010) were significant factors associated with mortality. The Kaplan-Meier analysis disclosed that AKI patients suffered lower cumulative survival than non-AKI patients (log-rank test, chi-square = 5.115, *P* = 0.024).

**Conclusions:**

AKI was common (66.0%) after methanol intoxication and was predictive of in-hospital hospital mortality. The development of AKI was associated with a 19.670-fold higher risk of in-hospital mortality.

## Background

Methanol poisoning is a serious public health issue in developing countries [1]. Methanol is gradually metabolized via alcohol dehydrogenase to formaldehyde, which is quickly metabolized to formate, which is responsible for toxicity [2]. The clinical course of methanol toxicity is characterized by the development of metabolic acidosis after a latent period, which is the time taken for methanol to be metabolized to formate. Later, there are various visual symptoms progressing to visual impairment, but some methanol cases could develop AKI, shock, multi-organ failure or mortality [1, 2].

In this study, we investigated the clinical features, spectrum and outcomes of AKI in patients with methanol intoxication, and most importantly, we evaluated the clinical predictors of in-hospital hospital mortality after methanol intoxication.

## Methods

### Patients

A total of 50 patients with methanol intoxication were seen at Chang Gung Memorial Hospital between 2000 and 2013.

### Inclusion and exclusion criteria

All patients aged 18 years and above were included in this study if they had a positive history of methanol exposure and their blood sample tested positive for methanol. Blood methanol level was examined by gas chromatography method [1]. Patients without identifiable blood methanol levels were excluded from this study.

### Detoxification protocols

Briefly, the protocols consisted of gastric lavage with normal saline, use of sodium bicarbonate, folic acid and ethanol antidote as described previously [1]. The indications for haemodialysis were [3]: severe metabolic acidosis, visual abnormality, deteriorating vital signs, AKI, electrolyte imbalance or blood methanol level of higher than 50 mg/dL.

### Haemodialysis

Haemodialysis was performed for 4 h via a temporary femoral catheter as described previously [1].

### Definition of AKI

AKI was defined as an abrupt (within 24–48 h) decrease in glomerular filtration rate due to renal damage that causes fluid and metabolic waste retention and alteration of electrolyte and acid-base balance [4, 5].

### Statistical analysis

The continuous variables were expressed as the means ± standard deviations for the numbers of observations, whereas the categorical variables were expressed as numbers (percentages). Non-normal distribution data were presented as medians (interquartile ranges). For comparisons between groups, Student’s *t*-test was used for quantitative variables, whereas the chi-square or Fisher’s exact test was used for categorical variables. Survival data were analysed with the Kaplan-Meier method and tested for significance using the log-rank test. A univariate binary logistic regression analysis was performed to compare the frequency of potential risk factors associated with mortality. The variables included acute kidney injury, age, anion gap, diabetes mellitus, ethanol level, glasgow coma scale score, habitual alcohol user, haemodialysis, hepatitis B or C virus carrier, hypertension, hypothermia, male, methanol level, osmolarity gap, pH, sodium bicarbonate, time from exposure to hospital arrival, time from exposure to haemodialysis initiation and unintentional exposure. To control for confounders, a stepwise backward multivariate binary logistic regression analysis was performed to analyse the variables that were significant on univariate analysis. The criterion for significance to reject the null hypothesis was a 95% confidence interval. The statistical analyses were performed using IBM SPSS Statistics Version 20 for Mac (IBM Corporation, Armonk, NY, USA).

## Results

Table 1 shows that most of the patients were middle-aged (47.8 ± 14.9 years), predominantly male (74.0%), and habitual alcohol consumers (70.0%). The majority of the incidents were oral exposures (96.0%) and unintentional (66.0%). Two (4.0%) patients attempted suicide by intravenous injection of methanol. Furthermore, consumption of illegal commercial alcohol products accounted for most cases (56.0%) of methanol intoxication. Notably, 5 (10.0%) patients suffered methanol intoxication after ingestion of methomyl pesticide that contained methanol as a solvent.

**Table 1:** Baseline characteristics of patients with methanol intoxication, stratified according to status of renal damage as AKI or non-AKI (*n* = 50)

Variable	AKI patients (*n* = 33)	Non-AKI patients (*n* = 17)	All patients (*N* = 50)	*P* value
Age, years	50.9 ± 13.7	41.6 ± 15.6	47.8 ± 14.9	0.034*
Male, n (%)	30 (90.9)	7 (42.8)	37 (74.0)	0.000***
Hypertension, n (%)	11 (33.3)	1 (5.9)	12 (24.0)	0.031*
Diabetes mellitus, n (%)	6 (18.2)	1 (5.9)	7 (14.0)	0.235
Hepatitis B or C virus carrier, n (%)	7 (21.2)	0 (0)	7 (14.0)	0.041*
Time from exposure to hospital arrival, hours	9.6 ± 17.8	3.5 ± 5.9	7.5 ± 15.1	0.180
Time from exposure to initiation of haemodialysis, hours	22.7 ± 18.4	12.0 ± 6.5	19.0 ± 16.0	0.051
Unintentional exposure, n (%)	27 (82.8)	6 (35.3)	33 (64.0)	0.001***
Habitual alcohol user, n (%)	28 (84.8)	7 (41.2)	35 (70.0)	0.001***
Route of exposure, n (%)				0.626
Oral exposure	32 (97.0)	16 (94.1)	48 (96.0)	
Intravenous exposure	1 (3.0)	1 (5.9)	2 (4.0)	
Source of methanol, n (%)				0.003**
Illegal commercial alcohol, n (%)	22 (66.7)	6 (35.3)	28 (56.0)	
Illegal handmade alcohol, n (%)	5 (15.2)	0 (0)	5 (10.0)	
Methomyl pesticide, n (%)	3 (9.1)	2 (11.8)	5 (10.0)	
Industrial methanol, n (%)	3 (9.1)	9 (52.9)	12 (24.0)	

*P < 0.05, **P < 0.01, and ***P < 0.001

Compared to non-AKI patients (Table 1), the AKI patients were older (50.9 ± 13.7 versus 41.6 ± 15.6 years, *P* = 0.034), predominantly male (90.9% versus 42.8%, *P* = 0.000), had higher proportions of hypertension (33.3% versus 5.9%, *P* = 0.031) and hepatitis B or C virus carriers (21.2% versus 0%, *P* = 0.041), had higher rates of unintentional exposure (82.8% versus 35.3%, *P* = 0.001), had more habitual alcohol use (84.8% versus 41.2%, *P* = 0.001) and had more consumption of illegal commercial alcohols (66.7% versus 35.5%, *P* = 0.003).

Table 2 shows that the latent periods of methanol intoxication were 5.3 ± 11.4 h and that symptoms of dyspnoea (60.0%), respiratory failure (52.0%), nausea/vomiting (42.0%), deep coma (36.0%), hypotension (32.0%), blurred vision (32.0%) and hypothermia (30.0%) were common. Moreover, there were more incidents of dyspnoea (75.8% versus 29.4%, *P* = 0.002) and respiratory failure (63.6% versus 29.4%, *P* = 0.022) in the AKI patients than in the non-AKI patients. In addition, the laboratory studies found that AKI patients suffered from more severe metabolic acidosis than non-AKI patients (Table 3). Nevertheless, none of the patients suffered from haemolysis or myoglobinuria.

**Table 2:** Clinical manifestations of patients with methanol intoxication, stratified according to status of renal damage as AKI or non-AKI (*n* = 50)

Variable	AKI patients (*n* = 33)	Non-AKI patients (*n* = 17)	All patients (*N* = 50)	*P* value
Latent period, hours	6.9 ± 13.2	2.4 ± 6.0	5.3 ± 11.4	0.191
Hypothermia, n (%)	12 (36.4)	3 (17.6)	15 (30.0)	0.171
Hypotension, n (%)	13 (39.4)	3 (17.6)	16 (32.0)	0.118
Bradycardia, n (%)	5 (15.2)	2 (11.8)	7 (14.0)	0.744
Blurred vision, n (%)	11 (33.3)	5 (29.4)	16 (32.0)	0.778
Blindness, n (%)	5 (15.2)	0 (0)	5 (10.0)	0.091
Photophobia, n (%)	1 (3.0)	1 (5.9)	2 (4.0)	0.626
Mydriasis, n (%)	5 (15.2)	1 (5.9)	6 (12.0)	0.339
Dyspnoea, n (%)	25 (75.8)	5 (29.4)	30 (60.0)	0.002**
Acute respiratory failure, n (%)	21 (63.6)	5 (29.4)	26 (52.0)	0.022*
Nausea/vomiting, n (%)	14 (42.4)	7 (41.2)	21 (42.0)	0.933
Gastrointestinal bleeding, n (%)	12 (36.4)	4 (23.5)	16 (32.0)	0.357
Abdominal pain, n (%)	10 (30.3)	3 (17.6)	13 (26.0)	0.334
Pancreatitis, n (%)	4 (12.1)	0 (0)	4 (8.0)	0.134
Hepatitis, n (%)	2 (6.1)	0 (0)	2 (4.0)	0.300
Glasgow coma scale score	9.5 ± 5.5	11.8 ± 5.2	10.3 ± 5.5	0.170
Deep coma, n (%)	14 (42.4)	4 (23.5)	18 (36.0)	0.187

*P < 0.05 and **P < 0.01

**Table 3:** Laboratory data at admission of patients with methanol intoxication, stratified according to status of renal damage as AKI or non-AKI (*N* = 50)

Variable	AKI patients (*n* = 33)	Non-AKI patients (*n* = 17)	All patients (*N* = 50)	*P* value
Blood urea nitrogen, mg/dL	22.4 ± 18.1	12.4 ± 4.3	18.8 ± 15.4	0.035*
Creatinine, mg/dL (admission)	2.51 ± 1.24	0.87 ± 0.17	1.97 ± 1.28	0.000***
Creatinine, mg/dL (peak)	3.23 ± 2.00	1.12 ± 0.94	2.54 ± 1.99	0.000***
Methanol level, mg/dL	33.1 ± 77.2	64.5 ± 75.5	43.8 ± 77.4	0.176
Ethanol level, mg/dL	48.6 ± 57.0	71.6 ± 125.3	56.4 ± 85.8	0.390
Arterial blood gas				
pH	7.055 ± 0.232	7.306 ± 0.190	7.141 ± 0.248	0.000***
pCO_2_, mmHg	26.5 ± 14.1	36.9 ± 11.1	30.0 ± 13.9	0.011*
pO_2_, mmHg	110.3 ± 60.0	112.8 ± 58.7	111.2 ± 59.0	0.890
Bicarbonate, mmol/L	8.7 ± 7.3	18.8 ± 6.8	12.2 ± 8.6	0.000***
Base excess, mmol/L	−17.9 ± 10.0	−7.4 ± 9.1	−13.5 ± 10.9	0.001**
Osmolarity, mOsm/kg H_2_O	341.0 ± 42.1	329.3 ± 26.0	336.9 ± 37.4	0.351
Osmolarity gap, mOsm/kg H_2_O	50.5 ± 84.2	37.3 ± 28.4	44.7 ± 65.0	0.624
Anion gap, mmol/L	33.4 ± 14.8	16.3 ± 7.3	27.2 ± 15.0	0.000***
Calcium, mEq/L	7.7 ± 0.9	7.5 ± 0.9	7.7 ± 0.9	0.526
Sodium, mEq/L	138.1 ± 6.1	141.7 ± 3.1	139.3 ± 5.5	0.029*
Potassium, mEq/L	4.6 ± 1.1	3.5 ± 0.6	4.2 ± 1.1	0.001*
Chloride, mEq/L	96.8 ± 8.9	106.9 ± 3.5	100.6 ± 8.8	0.000***
Amylase, mg/dL	137.8 ± 84.0	294.3 ± 477.4	182.5 ± 250.7	0.310
Lipase, mg/dL	179.1 ± 206.4	39.5 ± 14.0	154.8 ± 194.4	0.199
Albumin, g/dL	3.05 ± 1.01	3.57 ± 0.69	3.26 ± 0.91	0.297
Aspartate aminotransferase, U/L	303.7 ± 507.1	50.3 ± 37.9	245.2 ± 455.6	0.240
Alanine aminotransferase, U/L	96.4 ± 122.1	32.0 ± 24.7	73.9 ± 103.6	0.060
Random glucose, mg/dL	223.6 ± 145.5	126.6 ± 34.1	183.0 ± 121.8	0.026*
White blood cell count, 1000/μL	16.2 ± 9.7	11.6 ± 6.1	14.6 ± 8.8	0.077
Haemoglobin, g/dL	13.2 ± 3.1	14.0 ± 1.6	13.5 ± 2.7	0.311
Platelet count, 1000/μL	192.2 ± 109.6	242.9 ± 68.4	209.4 ± 99.8	0.089

*P < 0.05, **P < 0.01, and ***P < 0.001

By the end of this study, 13 (39.5%) AKI patients and 1 (5.9%) non-AKI patient had died. The overall in-hospital hospital mortality rate was 28% (Table 4).

**Table 4:** Outcome of patients with methanol intoxication, stratified according to status of renal damage as AKI or non-AKI (*n* = 50)

Variable	AKI patients (*n* = 33)	Non-AKI patients (*n* = 17)	All patients (*N* = 50)	*P* value
Gastric lavage, n (%)	22 (66.7)	7 (41.2)	29 (58.0)	0.084
Endotracheal intubation, n (%)	21 (63.6)	5 (29.4)	26 (52.0)	0.022*
Inotropic agent infusion, n (%)	13 (39.4)	3 (17.6)	16 (32.0)	0.118
Sodium bicarbonate, n (%)	27 (81.8)	7 (41.2)	34 (68.0)	0.004**
Ethanol, n (%)	13 (39.4)	8 (47.1)	21 (42.0)	0.603
Fomepizole, n (%)	0 (0)	0 (0)	0 (0)	1.000
Folic acid, n (%)	18 (54.5)	8 (47.1)	26 (52.0)	0.616
Haemodialysis, n (%)	24 (72.7)	13 (76.5)	37 (74.0)	0.775
Duration of hospitalization, day	9.5 ± 9.1	8.8 ± 8.0	9.2 ± 8.7	0.785
In-hospital mortality, n (%)	13 (39.4)	1 (5.9)	14 (28.0)	0.012*

*P < 0.05 and **P < 0.01

**Fig. 1:**
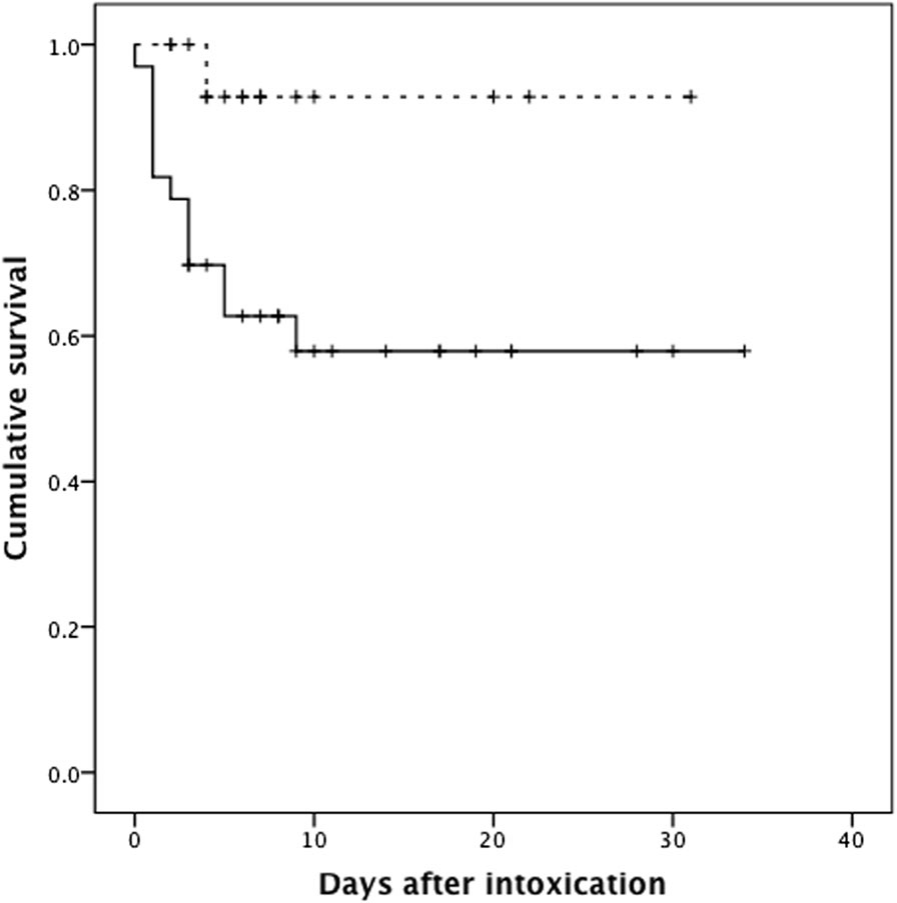
Kaplan-Meier analysis. AKI patients (solid line) suffered from lower cumulative survival than non-AKI patients (dashed line) (log-rank test, chi-square = 5.115, *P* = 0.024)

In a multivariate binary logistic regression model (Table 5), it was demonstrated that AKI (odds ratio 19.670, confidence interval 1.026–377.008, *P* = 0.048) and Glasgow coma scale score (odds ratio 1.370, confidence interval 1.079–1.739, *P* = 0.010) were significant factors associated with mortality. The presence of AKI was associated with a 19.670-fold higher risk of in-hospital mortality. Finally, the Kaplan-Meier analysis disclosed that AKI patients suffered lower cumulative survival than did non-AKI patients (Fig. 1) (log-rank test, chi-square = 5.115, *P* = 0.024).

**Table 5:** A binary logistic regression model for analysis of mortality (*N* = 50)

Variable	Univariate analysis	*P* value	Multivariate analysis	*P* value
	Odds ratio (95% confidence interval)		Odds ratio (95% confidence interval)	
Acute kidney injury (yes)	10.400 (1.227–88.178)	0.032*	19.670 (1.026–377.008)	0.048*
Age (each increase of 1 year)	1.044 (0.997–1.093)	0.070		
Anion gap (each increase of 1 mmol/L)	1.025 (0.980–1.072)	0.275		
Diabetes mellitus (yes)	1.033 (0.176–6.067)	0.971		
Ethanol level (each increase of 1 mg/dL)	0.996 (0.989–1.004)	0.324		
Glasgow coma scale score (each decrease of 1 score)	1.420 (1.171–1.721)	0.000***	1.370 (1.079–1.739)	0.010*
Habitual alcohol user (yes)	1.833 (0.429–7.836)	0.413		
Haemodialysis (yes)	0.833 (0.209–3.323)	0.796		
Hepatitis B or C virus carrier (yes)	2.182 (0.421–11.318)	0.353		
Hypertension (yes)	2.302 (0.585–9.056)	0.233		
Hypothermia (yes)	15.500 (3.474–69.159)	0.000***	6.905 (0.724–65.873)	0.093
Male (yes)	2.640 (0.504–13.835)	0.251		
Methanol level (each increase of 1 mg/dL)	1.003 (0.993–1.012)	0.598		
Osmolarity gap (each increase of 1 mOsm/kg H_2_O)	1.016 (0.997–1.036)	0.101		
pH (each decrease of 1 unit)	59.981 (3.074–878.999)	0.006**	3.981 (0.061–258.848)	0.517
Sodium bicarbonate (yes)	0.262 (0.051–1.350)	0.109		
Time from exposure to hospital arrival (each increase of 1 h)	1.034 (0.970–1.101)	0.306		
Time from exposure to haemodialysis initiation (each increase of 1 h)	1.001 (0.956–1.049)	0.954		
Unintentional exposure (yes)	1.413 (0.368–5.419)	0.614		

*P < 0.05, **P < 0.01, and ***P < 0.001

## Discussion

The overall in-hospital mortality rate was 28.0, and 66.0% of these patients suffered from AKI. These figures were comparable with data from other poison centres. As shown in Table 6, the published AKI and mortality rates were 15.4–66.0% and 0–48.0%, respectively [1, 6–25]. Therefore, patients with AKI should be recognized early and aggressively treated to avoid severe complications or mortality.

**Table 6:** Comparison of AKI and mortality rates between current and published studies (sample size ≥10)

Study	Year	Area	Sample size, n	Methanol level, mg/dL	AKI rate, %	Mortality rate, %
Liu et al. [6]	1998	Canada	50			36.0
Meyer et al. [7]	2000	America	24			33.3
Verhelst et al. [8]	2004	Belgium	25		60.0	24.0
Hovda et al. [9]	2005	Norway	51	80.0		17.6
Hassanian-Moghaddam et al. [10]	2007	Iran	25			48.0
Paasma et al. [11]	2007	Estonia	154			44.0
Brahmi et al. [12]	2007	Tunisia	16	140.0		19.0
Rzepecki et al. [13]	2012	Polish	288	50.1		3.8
Paasma et al. [14]	2012	Norway, Estonia, Tunisia, Iran	203	140.6		23.6
Shah et al. [15]	2012	India	63			31.7
Kute et al. [16]	2012	India	91			3.3
Massoumi et al. [17]	2012	Iran	51			7.8
Desai et al. [18]	2013	India	122	15.9		8.2
Sanaei-Zadeh et al. [19]	2013	Iran	42			40.5
Salek et al. [20]	2014	Czech	13	143.0	15.4	0
Zakharov et al. [21]	2014	Czech	121	86.9		33.9
Lee et al. [1]	2014	Taiwan	32	121.9	59.4	34.4
Lachance et al. [22]	2015	Canada	55	200.0		1.8
Rostrup et al. [23]	2016	Libya; Kenya	1066; 467			9.5; 26.9
Collister et al. [24]	2017	Canada	10	23.5		
Rulisek et al. [25]	2017	Czech	106	27.8		21.7
Current study	2018	Taiwan	50	43.8	66.0	28.0

AKI is a life-threatening complication that is associated with high death rates in intoxicated patients. The main aetiologies of AKI are ischaemia, hypoxia, or nephrotoxicity [26]. In cases of methanol intoxication, AKI has been reported, but limited studies have been performed to study this renal outcome. Although Salek et al. [20] found that only 2 of 13 (15.4%) methanol patients developed AKI, our previous analysis [1] indicated that AKI is common (19 of 32 or 59.4%) after methanol exposure. Similarly, Verhelst et al. [8] found that AKI developed in 15 of 25 (60.0%) patients with methanol intoxication. Compared with 10 non-AKI patients, the 15 AKI patients had a lower blood pH value on admission, a higher serum osmolality, and a higher peak formate concentration. According to Verhelst’s study [8], the aetiologies of methanol nephrotoxicity may be due to direct factors, such as high blood methanol and formate concentrations, or indirect factors, such as haemolysis and myoglobinuria [8].

Nevertheless, the aetiologies of AKI in our patients remained uncertain. In contrast to Verhelst’s hypothesis, none of the patients suffered from haemolysis or myoglobinuria. There were more incidents of respiratory failure (*P* = 0.022) in the AKI group than in the non-AKI group. These patients were intubated and receiving mechanical ventilator support. Previous studies [27, 28] have demonstrated that AKI can be induced by acute lung injury, which occurs because lung damage releases inflammatory mediators into the bloodstream that can affect renal function. According to a meta-analysis study [29], endotracheal intubation is associated with a threefold increase in the odds of developing AKI. Compared to non-AKI patients, the AKI patients were also older (*P* = 0.034) and had higher proportions of hypertension (*P* = 0.031). The association between age and hypertension is not surprising. As pointed out previously [30], many clinical circumstances could predispose a patient to progress with AKI, including age, sepsis, operation, and comorbidities, such as hypertension, diabetes mellitus, cardiovascular disease, malignancy, and chronic kidney disease.

The analysis indicates that AKI was associated with a higher risk of in-hospital death. In a multivariate binary logistic regression model, it was demonstrated that AKI was a significant factor associated with mortality (*P* = 0.048, Table 5). Kaplan-Meier analysis also revealed AKI patients suffered lower cumulative survival than non-AKI patients (*P* = 0.024) (Fig. 1). Clinical evidence suggests that AKI not only is an indicator for severity of illness but also leads to earlier onset of multi-organ dysfunction with profound effects on mortality rates [31]. In laboratory studies, it is demonstrated that AKI is not an isolated event; it engenders remote organ injury through a series of events that involves pro-inflammatory cytokine release, oxidative stress, immune cell stimulation, leukocyte extravasation, endothelial cell damage and vessel permeability leading to tissue oedema development [31, 32]. Our previous studies also revealed that AKI predicts mortality after intoxications, such as paraquat [5] or charcoal burning [33] suicide.

The foundation of treatment for methanol intoxication is the administration of an antidote, which blocks the function of alcohol dehydrogenase, thereby preventing the formation of toxic metabolites [34]. There are two antidotes: ethanol (a competitive alcohol dehydrogenase substrate) and fomepizole (an alcohol dehydrogenase inhibitor), which can be administered to block alcohol dehydrogenase metabolism. Nevertheless, none of our patients received fomepizole therapy because this drug was not available at our hospital (Table 4).

Five (10.0%) patients suffered methanol intoxication after ingestion of methomyl pesticide that contained methanol as a solvent (Table 1). The clinical findings observed in these cases were similar to a previous outbreak of foodborne illness due to methomyl pesticide intoxication in Korea [35]. It is possible that the combined toxicity of methomyl pesticide and methanol solvent was responsible for the symptoms. Methomyl pesticide is exceptionally toxic if ingested [36]. It is a carbamate insecticide and can induce acute cholinergic crisis by reversible inhibition of cholinesterase [37]. To minimize health impacts, the United States Environmental Protection Agency has classified methomyl products used in agricultural settings as “restricted use”, meaning that they can be used only by or under the supervision of certified farmers [36].

Two (4.0%) patients attempted suicide by intravenous injection of methanol (Table 1). Their blood methanol concentrations were 71.2 mg/dL and 5.0 mg/dL. Both patients were successfully treated with haemodialysis without any complications. Few human data exist in the literature regarding the outcome of intravenous methanol poisoning, although the methanol extraction residue of Bacillus Calmette-Guerin could be safely injected into patients with advanced cancer by the intravenous route without causing complications [38]. Nevertheless, the administered amount was very low under that circumstance. Wang et al. [39] reported a human case of intravenous methanol intoxication in 1997. Ophthalmologic examination on the seventh day disclosed hyperaemia of the optic disc with peripapillary haemorrhage and cotton-wool spots. The severity of retina injury was caused by 100% bioavailability of methanol after intravenous injection and lack of first-pass metabolism [39]. In addition, the patient arrived at the hospital too late (after 7 days) to take advantage of detoxification procedures. On the other hand, the good prognosis of the current 2 patients depends on early hospital arrival, prompt diagnosis of methanol intoxication and speedy initiation of haemodialysis.

## Conclusions

AKI was common (66.0%) after methanol intoxication and was predictive of in-hospital mortality. The development of AKI was associated with a 19.670-fold higher risk of in-hospital mortality. Therefore, patients with AKI should be recognized early and aggressively treated to avoid mortality. Nevertheless, the retrospective nature of the study, small sample size, short follow-up duration, and absence of pre-admission serum creatinine and urine output measurements limit the certainty of our conclusions.

## Data Availability

The datasets used and/or analysed during the current study are available from the corresponding author on reasonable request. Furthermore, not only were all data securely protected (by delinking identifying information from the main data sets) and made available only to investigators, but they were also analysed anonymously.

## References

[1] Lee CY, Chang EK, Lin JL, Weng CH, Lee SY, Juan KC, Yang HY, Lin C, Lee SH, Wang IK (2014). Risk factors for mortality in Asian Taiwanese patients with methanol poisoning. Ther Clin Risk Manag.

[2] Jacobsen D, McMartin KE (1986). Methanol and ethylene glycol poisonings. Mechanism of toxicity, clinical course, diagnosis and treatment. Med Toxicol.

[3] Barceloux DG, Bond GR, Krenzelok EP, Cooper H, Vale JA (2002). American Academy of clinical toxicology practice guidelines on the treatment of methanol poisoning. J Toxicol Clin Toxicol.

[4] Andreucci M, Faga T, Pisani A, Perticone M, Michael A (2017). The ischemic/nephrotoxic acute kidney injury and the use of renal biomarkers in clinical practice. Eur J Intern Med.

[5] Weng CH, Chen HH, Hu CC, Huang WH, Hsu CW, Fu JF, Lin WR, Wang IK, Yen TH (2017). Predictors of acute kidney injury after paraquat intoxication. Oncotarget.

[6] Liu JJ, Daya MR, Carrasquillo O, Kales SN (1998). Prognostic factors in patients with methanol poisoning. J Toxicol Clin Toxicol.

[7] Meyer RJ, Beard ME, Ardagh MW, Henderson S (2000). Methanol poisoning. N Z Med J.

[8] Verhelst D, Moulin P, Haufroid V, Wittebole X, Jadoul M, Hantson P (2004). Acute renal injury following methanol poisoning: analysis of a case series. Int J Toxicol.

[9] Hovda KE, Hunderi OH, Tafjord AB, Dunlop O, Rudberg N, Jacobsen D (2005). Methanol outbreak in Norway 2002-2004: epidemiology, clinical features and prognostic signs. J Intern Med.

[10] Hassanian-Moghaddam H, Pajoumand A, Dadgar SM, Shadnia S (2007). Prognostic factors in methanol poisoning. Hum Exp Toxicol.

[11] Paasma R, Hovda KE, Tikkerberi A, Jacobsen D (2007). Methanol mass poisoning in Estonia: outbreak in 154 patients. Clin Toxicol (Phila).

[12] Brahmi N, Blel Y, Abidi N, Kouraichi N, Thabet H, Hedhili A, Amamou M (2007). Methanol poisoning in Tunisia: report of 16 cases. Clin Toxicol (Phila).

[13] Rzepecki J, Krakowiak A, Fiszer M, Czyzewska S, Winnicka R, Kolacinski Z, Politanski P, Swiderska S (2012). Acute methanol poisoning among patients of toxicology unit, Nofer Institute of Occupational Medicine in Lodz, during the period 2000-2009. Przegl Lek.

[14] Paasma R, Hovda KE, Hassanian-Moghaddam H, Brahmi N, Afshari R, Sandvik L, Jacobsen D (2012). Risk factors related to poor outcome after methanol poisoning and the relation between outcome and antidotes--a multicenter study. Clin Toxicol (Phila).

[15] Shah S, Pandey V, Thakore N, Mehta I (2012). Study of 63 cases of methyl alcohol poisoning (hooch tragedy in Ahmedabad). J Assoc Physicians India.

[16] Kute VB, Godara SM, Shah PR, Gumber MR, Goplani KR, Vanikar AV, Munjappa BC, Patel HV, Trivedi HL (2012). Hemodialysis for methyl alcohol poisoning: a single-center experience. Saudi J Kidney Dis Transpl.

[17] Massoumi G, Saberi K, Eizadi-Mood N, Shamsi M, Alavi M, Morteza A (2012). Methanol poisoning in Iran, from 2000 to 2009. Drug Chem Toxicol.

[18] Desai T, Sudhalkar A, Vyas U, Khamar B (2013). Methanol poisoning: predictors of visual outcomes. JAMA Ophthalmol.

[19] Sanaei-Zadeh H, Emamhadi M, Farajidana H, Zamani N, Amirfarhangi A (2013). Electrocardiographic manifestations in acute methanol poisoning cannot predict mortality. Arh Hig Rada Toksikol.

[20] Salek T, Humpolicek P, Ponizil P (2014). Metabolic disorders due to methanol poisoning. Biomed Pap Med Fac Univ Palacky Olomouc Czech Repub.

[21] Zakharov S, Pelclova D, Urban P, Navratil T, Diblik P, Kuthan P, Hubacek JA, Miovsky M, Klempir J, Vaneckova M (2014). Czech mass methanol outbreak 2012: epidemiology, challenges and clinical features. Clin Toxicol (Phila).

[22] Lachance P, Mac-Way F, Desmeules S, De Serres SA, Julien AS, Douville P, Ghannoum M, Agharazii M (2015). Prediction and validation of hemodialysis duration in acute methanol poisoning. Kidney Int.

[23] Rostrup M, Edwards JK, Abukalish M, Ezzabi M, Some D, Ritter H, Menge T, Abdelrahman A, Rootwelt R, Janssens B (2016). The methanol poisoning outbreaks in Libya 2013 and Kenya 2014. PLoS One.

[24] Collister D, Duff G, Palatnick W, Komenda P, Tangri N, Hingwala J (2017). A methanol intoxication outbreak from recreational ingestion of fracking fluid. Am J Kidney Dis.

[25] Rulisek J, Balik M, Polak F, Waldauf P, Pelclova D, Belohlavek J, Zakharov S (2017). Cost-effectiveness of hospital treatment and outcomes of acute methanol poisoning during the Czech Republic mass poisoning outbreak. J Crit Care.

[26] Basile DP, Anderson MD, Sutton TA (2012). Pathophysiology of acute kidney injury. Compr Physiol.

[27] Domenech P, Perez T, Saldarini A, Uad P, Musso CG (2017). Kidney-lung pathophysiological crosstalk: its characteristics and importance. Int Urol Nephrol.

[28] Husain-Syed F, Slutsky AS, Ronco C (2016). Lung-kidney cross-talk in the critically ill patient. Am J Respir Crit Care Med.

[29] van den Akker JP, Egal M, Groeneveld AB (2013). Invasive mechanical ventilation as a risk factor for acute kidney injury in the critically ill: a systematic review and meta-analysis. Crit Care.

[30] Yokota LG, Sampaio BM, Rocha EP, Balbi AL, Sousa Prado IR, Ponce D (2018). Acute kidney injury in elderly patients: narrative review on incidence, risk factors, and mortality. Int J Nephrol Renovasc Dis.

[31] Yap SC, Lee HT (2012). Acute kidney injury and extrarenal organ dysfunction: new concepts and experimental evidence. Anesthesiology.

[32] Druml W (2014). Systemic consequences of acute kidney injury. Curr Opin Crit Care.

[33] Chen YC, Tseng YC, Huang WH, Hsu CW, Weng CH, Liu SH, Yang HY, Chen KH, Chen HL, Fu JF (2016). Acute kidney injury predicts mortality after charcoal burning suicide. Sci Rep.

[34] Beatty L, Green R, Magee K, Zed P (2013). A systematic review of ethanol and fomepizole use in toxic alcohol ingestions. Emerg Med Int.

[35] Gil HW, Jeong MH, Park JS, Choi HW, Kim SY, Hong SY (2013). An outbreak of food borne illness due to methomyl pesticide intoxication in Korea. J Korean Med Sci.

[36] Web address: https://www.epa.gov/ingredients-used-pesticide-products/methomyl. Accessed 27 May 2019.

[37] Tsai MJ, Wu SN, Cheng HA, Wang SH, Chiang HT (2003). An outbreak of food-borne illness due to methomyl contamination. J Toxicol Clin Toxicol.

[38] Robinson E, Bartal A, Honigman J, Cohen Y (1977). A preliminary study of intravenous methanol extraction residue of BCG in treatment of advanced cancer. Br J Cancer.

[39] Wang JY, Lin YF, Lin SH (1999). Methanol intoxication with retinal injury by intravenous injection. Ann Emerg Med.

